# Treating seizures and epilepsy with anticoagulants?

**DOI:** 10.3389/fncel.2013.00019

**Published:** 2013-03-05

**Authors:** Nicola Maggio, Ilan Blatt, Andreas Vlachos, David Tanne, Joab Chapman, Menahem Segal

**Affiliations:** ^1^Talpiot Medical Leadership Program, The Chaim Sheba Medical CenterTel HaShomer, Israel; ^2^Department of Neurology, The J. Sagol Neuroscience Center, The Chaim Sheba Medical CenterTel HaShomer, Israel; ^3^ Department of Neurology, Sackler Faculty of Medicine, Tel Aviv UniversityTel Aviv, Israel; ^4^Institute of Clinical Neuroanatomy, Neuroscience Center, Goethe-University FrankfurtFrankfurt/Main, Germany; ^5^Department of Neurobiology, The Weizmann Institute of ScienceRehovot, Israel

**Keywords:** thrombin, PAR-1, seizures, blood–brain barrier, novel anticoagulants

## Abstract

Thrombin is a serine protease playing an essential role in the blood coagulation cascade. Recent work, however, has identified a novel role for thrombin-mediated signaling pathways in the central nervous system. Binding of thrombin to protease-activated receptors (PARs) in the brain appears to have multiple actions affecting both health and disease. Specifically, thrombin has been shown to lead to the onset of seizures via PAR-1 activation. In this perspective article, we review the putative mechanisms by which thrombin causes seizures and epilepsy. We propose a potential role of PAR-1 antagonists and novel thrombin inhibitors as new, possible antiepileptic drugs.

## THROMBIN SIGNALING IN THE BRAIN

Thrombin is a serine protease, which plays an essential role in the blood coagulation cascade (Siller-Matula et al., 2011). Upon its formation following the enzymatic cleavage of prothrombin by activated Factor X, thrombin regulates a cascade of proteolytic events ultimately leading to the formation of blood clots (Lippi et al., 2012). Lately, however, novel signaling cascades mediated by thrombin have been discovered (Siller-Matula et al., 2011). Specifically, through the activation of the protease-activated receptors (PARs), thrombin seems to directly affect the activity of multiple cell types and regulate a variety of biological functions, such as inflammation, leukocyte migration, cellular proliferation, vascular permeability and tone, edema formation, and other processes related to tissue repair (Coughlin, 2000, 2001; Sambrano et al., 2001; Chen and Dorling, 2009; Schuepbach et al., 2009; Spiel et al., 2011).

Protease-activated receptors belong to a unique family of G protein-coupled receptors (Luo et al., 2007). Their activation is initiated by an irreversible site-specific proteolytic cleavage in the N-terminal extracellular region. The uncovered N-terminal region then acts as a tethered ligand which activates the receptor (Gingrich and Traynelis, 2000). PARs are expressed in the brain and while PAR-2 represents a class of trypsin/tryptase-activated receptors, PAR-1, PAR-3, and PAR-4 are most effectively activated by thrombin (Gingrich and Traynelis, 2000). In the brain, PAR-1 has been detected in both neurons and astrocytes, with the latter demonstrating stronger immunoreactivity in human brain tissue (Junge et al., 2004). High levels of PAR-1 are detected in the hippocampus, cortex, and striatum of humans (Junge et al., 2004). While the molecular pathways activated by PAR-1 in neurons are yet under investigation, in the brain PAR-1 activation has been shown to modulate synaptic transmission and plasticity through the enhancement of *N*-methyl-D-aspartate (NMDA) receptor (NMDAR) currents (Gingrich et al., 2000; Lee et al., 2007; Maggio et al., 2008). In addition, PAR-1 knockout animals present profound deficits in hippocampus-dependent learning and memory processes (Almonte et al., 2007, 2013). Altogether, it seems that PAR-1 plays a critical role in memory formation and synaptic plasticity.

Interestingly, a variety of pathological conditions have been associated with changes in the expression of PAR-1 in the brain. In Parkinson's disease, a significant increase in the number of astrocytes expressing PAR-1 has been reported in the substantia nigra pars compacta (Ishida et al., 2006). In addition, upregulation of PAR-1 in astrocytes has been observed in HIV encephalitis, (Boven et al., 2003) indicating that this receptor might be implicated in the pathogenesis of neuroinflammation. This idea is supported by the evidence of elevated levels of thrombin in an experimental model of multiple sclerosis (Beilin et al., 2005) as well as in other inflammatory brain diseases (Chapman, 2006). Stimulation of PAR-1 by thrombin causes proliferation of glia and potentially produces reactive gliosis, infiltration of inflammatory cells, and angiogenesis (Striggow et al., 2001). Finally, expression of PAR-1 is increased in experimental models of Alzheimer's disease (Pompili et al., 2004) and brain ischemia (Striggow et al., 2001).

## THROMBIN CAUSES SEIZURES AND EPILEPSY THROUGH PAR-1 ACTIVATION

Serine proteases are normally expressed in the brain at very low level (Luo et al., 2007). Nevertheless, their concentration can increase abnormally following the breakdown of the blood–brain barrier (BBB). Under this scenario, a large, non-selective increase in the permeability of brain capillaries and tight junctions takes place, allowing the entry of high molecular weight proteins (Ballabh et al., 2004) and blood components into the cerebral tissue. This event can occur under several neurological conditions (Ballabh et al., 2004; Tomkins et al., 2007), particularly after hemorrhagic/ischemic stroke (Hjort et al., 2008; Bang et al., 2009) or traumatic brain injury (TBI; Barzo et al., 1997; Tomkins et al., 2008). Although there is a paucity of information concerning the amount of thrombin crossing the BBB, it has been demonstrated that thrombin levels increase more than 200-fold (from 100 pM to 25 nM) in the cerebrospinal fluid of patients with subarachnoid cerebral hemorrhage (Suzuki et al., 1992). In addition, when the bleeding occurs directly within the brain tissue, active thrombin and other proteases can freely diffuse into the brain parenchyma until clotting closes off the injured vessels. In this respect, our preliminary data suggest that under experimental conditions, depletion of thrombin from the clot appears to be continuous, with the concentration of thrombin in cerebrospinal fluid increasing several-folds over a 24-h time window.

**FIGURE 1:**
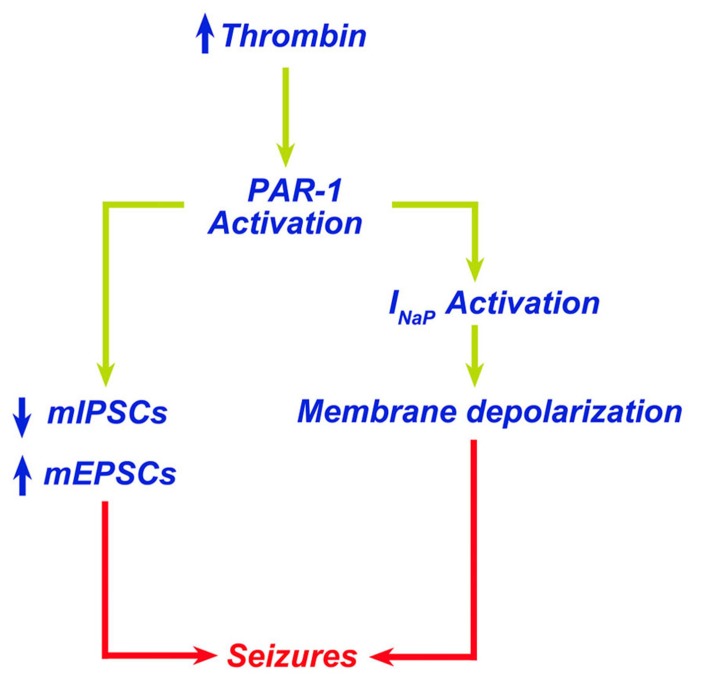
**Thrombin leads to seizures through PAR-1 activation.** Increased levels of thrombin in the brain lead to PAR-1 activation. PAR-1 promotes membrane depolarization due to activation of *I*_Nap_ and altered balance of excitatory/inhibitory synapses leading to seizures.

A direct consequence of the contact of thrombin with the brain tissue is the onset of seizures. Lee et al. (1997) reported that intracerebral injections of thrombin resulted in focal motor seizures. Interestingly, thrombin injected together with its inhibitor alpha-(2-naphthylsulfonyl-glycyl)-4-amidinophenylalanine piperidide (alpha-NAPAP) did not cause any sign of either clinical or electrographic seizures (Lee et al., 1997). Similarly, mice engineered to lack protein Nexin-1, an endogenous thrombin inhibitor, have an increased susceptibility to kainic acid-induced seizures (Luthi et al., 1997). Our own work has demonstrated that thrombin-induced seizures are mediated by activation of PAR-1 (Maggio et al., 2008). In hippocampal slices, thrombin at a concentration of 5 nM (1 U/ml) increases spontaneous firing of CA3 pyramidal cells (Maggio et al., 2008). In order to examine whether thrombin facilitates the onset of epileptic discharges in conditions mimicking a BBB breakdown in the slice (Chen and Swanson, 2003; Beart and O'Shea, 2007), we exposed neurons to thrombin in presence of elevated [K^+^]_o_ or low levels of glutamate. In normal slices, addition of 4 mM K^+^ did not produce any noticeable spontaneous seizures, which were clearly seen when [K^+^] were raised by 15 mM. Similarly, 500 μM but not 100 μM glutamate produced spontaneous seizure-like activity in the slice. Strikingly, thrombin facilitated the response to the lower concentration of K^+^ (4 mM) and glutamate (100 μM) to produce seizure-like activity. This activity was mediated by PAR-1 activation, since it was mimicked by a peptide agonist of the receptor and blocked by its antagonist SCH79797. Interestingly, the facilitatory action of thrombin on the production of seizure-like activity did not depend on NMDARs as it was not affected by the selective NMDAR-inhibitors ifenprodil or by 2R-amino-5-phosphonovaleric acid (APV). Recently, Isaeva et al. (2012) exposed hippocampal slices from immature rats (P6 to P15) to large concentrations of thrombin (10 U/ml) to find that thrombin depolarized membrane potential of neurons and produced a hyperpolarizing shift of tetrodotoxin-sensitive *I*_Nap_ through a PAR-1-mediated mechanism (Isaeva et al., 2012). In addition, we have reported that thrombin affects synaptic transmission in hippocampal CA3 neurons by enhancing both frequency and amplitude of mEPSCs while reducing frequency and amplitude of mIPSCs (Maggio et al., 2012). Taken together, these studies clearly indicate a proepileptic effect of thrombin which upon PAR-1 activation induces membrane and synaptic changes leading to seizures (**Figure 1**). Less clear, however, is whether the thrombin-induced increase in neuronal activity could lead to epilepsy later on. Indeed, it has been demonstrated that high concentrations of thrombin, usually reached in pathological settings following thrombin extravasation into the brain, may induce apoptosis (Lee da et al., 2006; Wang et al., 2006; Xi et al., 2006; Luo et al., 2007). Accordingly, thrombin-induced neuronal cell loss could lead to circuit reorganization and onset of epilepsy. While circuit reorganization following injury is a well known cause of hyper excitability (Heinemann et al., 2002; Heinemann, 2004), no information is currently available on the possible role of thrombin and PAR-1 activation in this situation.

## PAR-1 ANTAGONISTS AND THROMBIN INHIBITORS AS NEW ANTIEPILEPTIC DRUGS?

**FIGURE 2:**
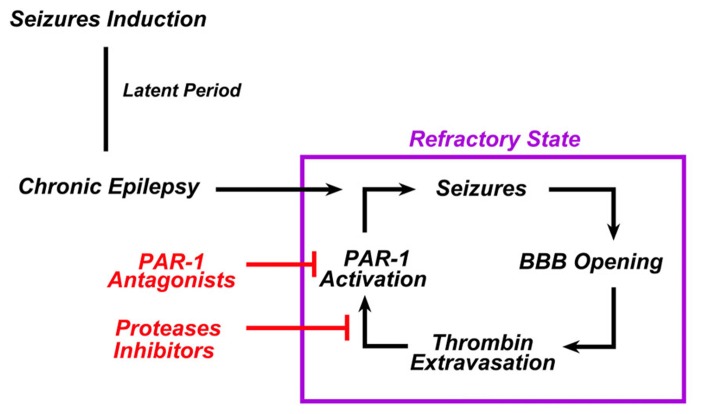
**In chronic epilepsy, thrombin constantly sustains the epileptic process.** In chronic epilepsy, recurrent seizures lead to the breakdown of BBB. Extravasation of thrombin from the blood into the brain tissue activates PAR-1 in the brain. PAR-1 activation sustains or even enhances the epileptogenic processes responsible for the refractoriness of such syndrome. The use of either PAR-1 antagonists and/or serine proteases inhibitors could break this vicious cycle and prevent the occurrence of refractoriness.

Seizures and epilepsy are commonly observed in conjunction with stroke, TBI, and central nervous system infections, all conditions known to result in compromised BBB function (Tomkins et al., 2001; Ballabh et al., 2004). Regional patterns of BBB breakdown have been described during epileptiform seizures induced in animal models by various convulsive agents (Nitsch and Klatzo, 1983). Following BBB breakdown, seizures result from the exposure of the brain to serum components such as thrombin due to the increased permeability of the BBB (Kelly, 2008). In fact, even in the absence of hemorrhage, BBB breakdown may activate the coagulation cascade leading to intracerebral generation of thrombin (Stein et al., 2002; Chodobski et al., 2011; Pisapia et al., 2012). In this setting an enduring BBB breakdown due to uncontrolled seizures may lead to a continuous leak of thrombin into the brain, which in turn sustains the epileptic process (**Figure 2**).

If thrombin indeed is the major reason for seizures in this condition, it is tempting to speculate that PAR-1 antagonists and/or thrombin inhibitors could act as potential antiepileptic drugs. PAR-1 antagonists are a class of drugs currently tested in the context of cardiovascular diseases (Ahn et al., 2003; Landis, 2007; Lee and Hamilton, 2012). They are non-peptide small molecular compounds which differ in their effectiveness to inhibit PAR-1 (Ahn et al., 2003). They have both anticoagulant (Wielders et al., 2007) and antiaggregant (Lee and Hamilton, 2012) properties. However, unlike a direct thrombin inhibitor, they are thought to have minimal bleeding side-effects due to the inability of blocking the enzymatic action of thrombin in the coagulation cascade. We (Maggio et al., 2008, 2012) and others (Isaeva et al., 2012) have indeed shown that PAR-1 antagonists block the proepileptogenic effects of thrombin *in vitro*, however, no data currently exist on the role of PAR-1 antagonists as antiepileptic drugs in animal models of epilepsy following BBB breakdown. Furthermore, it is not known whether thrombin and PAR-1 levels are increased in the brains of experimental animals undergoing chronic epilepsy. In this context, our preliminary data based on Li^2^^+^- pilocarpine treated animals do show that this might indeed be the case. Interpretation of clinical data might provide important insights as well. Cardiac surgery has been associated with a high rate of seizures in the post-operative settings (Goldstone et al., 2011a,b; Hervey-Jumper et al., 2011). Thus, are patients treated with PAR-1 antagonists as prevention to reduce the prothrombotic risk occurring in cardiothoracic surgery (Landis, 2007) going to show less seizures in their post-operative outcomes?

Thrombin is the target of a newly developed class of anticoagulants proposed as alternatives to vitamin K-dependent anticoagulants for the prevention of stroke and systemic embolism in patients with atrial fibrillation (Zikria and Ansell, 2009; Dentali et al., 2012; Haft, 2012). Do these molecules have an antiepileptic activity as well? In this respect, the novel direct thrombin inhibitor, dabigatran, which blocks the proteolytic activity of thrombin, might mimic the antiepileptic effect of alpha-NAPAP by preventing the thrombin-dependent activation of PAR-1. A similar result, using a different mechanism may be achieved by the novel direct Factor X inhibitors, apixaban and rivaroxaban, which halt Factor X from converting prothrombin to thrombin. Currently, no clinical and experimental data are available to analyze whether these molecules might have a role in the prevention of seizures and epilepsy upon brain exposure to high intracerebral concentrations of thrombin. Indeed, it might be interesting to evaluate whether patients taking direct thrombin inhibitors for secondary stroke prevention are less prone to the development of seizures, a known complication of stroke. This being the case, our approach justifies the use of these drugs as more advantageous compared to the old vitamin K-dependent anticoagulants. A possible disadvantage for the use of these drugs in epileptic patients might be related to the increased risk of bleeding in people experiencing recurrent falls due to seizures. This is certainly possible, however, studies are needed to evaluate whether the dosage required for full anticoagulation are similar to the ones needed to reach an antiepileptic effect: thrombin concentrations are far higher in the serum (Siller-Matula et al., 2011) than in the brain following BBB breakdown (Striggow et al., 2000). The use of these drugs might as well be proposed in patients which may develop epilepsy following brain hemorrhage. This should however be very carefully considered by taking into account the type and modalities of the bleeding as well as possible continuation of anticoagulation in a population of patients at risk of recurrence.

In conclusions, work from recent years has disclosed a novel and clinically highly relevant role for thrombin in the pathogenesis of hyperexcitability in neuronal networks and the development of seizures and epilepsy. However, more data are needed to evaluate the precise role of thrombin in epilepsy. The identification of the thrombin-PAR-1 pathway playing a fundamental role in the pathophysiology of epilepsy may lead to the development of new therapeutic strategies and provide a rationale for testing PAR-1 antagonists and/or thrombin inhibitors in animal models of epilepsy.

## Conflict of Interest Statement

The authors declare that the research was conducted in the absence of any commercial or financial relationships that could be construed as a potential conflict of interest.
